# Time to Move: A 4‐Week Gamified Mobile Application Intervention to Promote Physical Activity in Secondary School Students

**DOI:** 10.1002/ejsc.70027

**Published:** 2025-07-25

**Authors:** Nimet Haşıl Korkmaz, Hasibe Çoruh

**Affiliations:** ^1^ Department of Physical Education and Sports Faculty of Sport Sciences Bursa Uludağ University Bursa Türkiye

**Keywords:** exercise, gamification, motivation, physical activity

## Abstract

This study aimed to help children and adolescents achieve their daily goal of 10,000 steps and daily exercise goals using the T2M (Time to Move) mobile gamification application. This study was a single‐blinding cluster‐randomised controlled trial conducted in a secondary school, including a control group (CG) and an experimental group (EG). The study consisted of a 1‐week familiarisation, 4‐week intervention and impact assessment session. Twenty‐five students aged 12–14 years (CG, *n* = 13 am EG, *n* = 12) participated. T2M mobile application was developed to track the students' daily step counts and exercises and increase their physical activity levels. Newfeel Onwalk One Plus pedometer was chosen to track the number of steps. In EG, gamification elements (badges, points and leaderboards) were used for students to achieve the goals, whereas in CG, these elements were not included. 40% of those in the control group completed the step goals and 15% completed the exercise goals. In comparison, 65% of the experimental group completed the step goals (***p* = 0.043** and ***d* = 0.40**) and 68% completed the exercise goals (***p* = 0.0001** and ***d* = 0.77**). Furthermore, the PAQ‐C scores were higher in the experimental group (***p* = 0.0114** and ***d* = 0.51**). As a result of the impact assessment session, it was determined that 70% of the students in the experimental group and 27% of those in the control group continued to exercise. It is observed that the experimental group completed more of the 10,000 steps per day and daily exercise goals. These findings suggest that T2M mobile‐based gamification application can increase the physical activity level of children and adolescents.

## Highlights

- The T2M mobile application uses gamification elements to promote physical activity among secondary school students.
- The experimental group achieved higher step (65%) and exercise (68%) goal completion rates compared to the control group (40% and 15%).
- A 4‐week intervention in the experimental group using the T2M gamified mobile application significantly increased students' daily step counts, exercise goal completion and PAQ‐C scores, indicating its effectiveness in enhancing physical activity and motivation among secondary school students.

## 1 Introduction

Regular physical activity provides many health benefits, including improved physical and mental health, reduced risk of obesity, cardiovascular and cardiometabolic health improvements and cognitive, social and psychological areas (Farooq et al. 2021; Alvarez‐Pitti et al. 2020; Poitras et al. 2016). Therefore, World Health Organization (WHO) guidelines recommend that children and adolescents engage in moderate‐to‐vigorous‐intensity PA (physical activity) for at least 60 min daily (WHO 2021; Bull et al. 2020). However, 80% of children and adolescents worldwide do not meet the WHO‐recommended PA guidelines (Aubert et al. 2022; Bull et al. 2020; Guthold et al. 2020). Physical inactivity continues into adulthood, increasing the risk of noncommunicable diseases such as obesity, diabetes and cardiovascular diseases (Cleven et al. 2020). Additionally, physical inactivity is a significant public health issue of the 21st century, ranking as the fourth leading cause of death globally, resulting in over 5 million deaths each year (Hallal et al. 2012; I.‐M. Lee et al. 2012; Blair 2009).

Childhood and adolescence are in critical periods when physical activity habits form, which usually continue into adulthood (TELAMA et al. 2014). Thus, innovative behaviour change interventions are needed to improve PA levels of children and adolescents in this age group. Gamification is an interactive way of setting goals and providing rewards to increase motivation and engagement in physical activity. Gamification is defined as using game design elements (such as points, leaderboard, progress bars and badges) in nongame contexts (such as education, marketing and healthcare) (Deterding et al. 2011). Gamification is a design strategy often used to encourage physical activity (Koivisto and Hamari 2019). According to comprehensive systematic reviews, gamified interventions positively affect physical activity levels (Mazeas et al. 2021; Yang et al. 2021).

The literature shows that gamified pedometer applications are frequently used to increase PA levels in children and adolescents. These are the gamified pedometer interventions ‘Strava, Pacer and MapMyWalk’ by Mateo‐Orcajada et al. (2023), ‘The StepSmart Challenge’ by Corepal et al. (2019) and ‘KIJANI’ by Willinger et al. (2024). However, pedometer interventions alone are insufficient for the additional benefits of physical activity. Performing some exercises is also recommended according to the WHO (2020) guidelines. However, many individuals have low motivation to integrate exercise into their daily lives. Therefore, it is observed that gamified exercise interventions are used. Among these, it is seen that ‘Fitocracy’ for adults and ‘Spirit50’ for the elderly use gamified exercise applications (Goh and Razikin 2015; Kappen et al. january 3, 2018). These gamification‐based applications are effective in motivating individuals to exercise. As can be seen from this current literature review, similar studies in recent years have primarily focused on physical activity (step count) or existing known exercise gamification apps that are not designed for children and adolescents. Also, to our knowledge, a mobile‐based gamification application that merges physical activity (step count) and exercise for children and adolescents has not been found in the literature. Thus, in this study, a mobile‐based gamification application specifically designed for children and adolescents—combining both step tracking and exercise components—was developed to increase their physical activity levels, step counts and exercise behaviours and its effects were examined.

In general, gamified interventions consist of a combination of game elements designed to encourage both intrinsic and extrinsic motivation. Gamification studies are based on various theories. Among these, self‐determination theory (SDT), developed by Ryan and Deci (2000), is one of the most widely used theories for understanding people's intrinsic and extrinsic motivations (Krath et al. 2021). SDT posits that individuals strive to satisfy three basic psychological needs: autonomy (a sense of control over one's actions), competence (a sense of expertise and success) and relatedness (a need to connect with others). When these needs are met, intrinsic motivation increases, leading to better participation and sustainable performance. Intrinsic motivation is shaped by the satisfaction and enjoyment one derives from the activity, whereas external rewards or outcomes influence extrinsic motivation. The T2M mobile‐based gamification application developed in our study aims to support individuals' intrinsic interests by providing real‐time physical activity and exercise data while increasing extrinsic motivation through a reward system. This approach strengthens the motivational foundation for increasing children's and adolescents' participation in physical activity.

### 1.1 This Study

This study aimed to investigate the effect of gamified and nongamified physical activity and exercise interventions of the T2M mobile application, which was created to encourage secondary school students to engage in physical activity and exercise, on their physical activity levels after 1 month. Additionally, we established the following research questions: (1) Is there a statistically significant difference between the control group (CG) and experimental group (EG) in achieving daily 10,000 steps and exercise goals for 4 weeks? (2) Is there a statistically significant difference between pretest and posttest results of the Physical Activity Questionnaire for Older Children (PAQ‐C) in the control group (CG) and experimental group (EG)?

## 2 Materials and Methods

### 2.1 Study Design and Intervention

**FIGURE 1:**
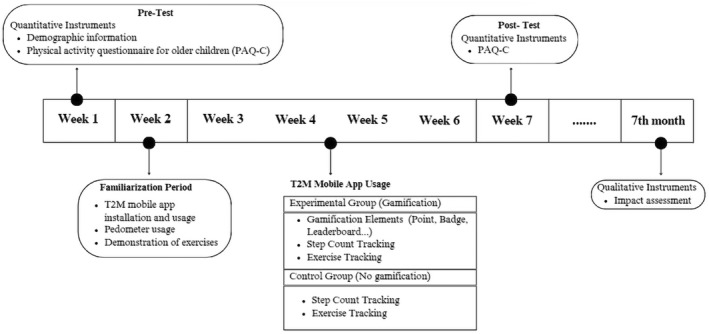
Research design.

Approval was received from the university's research ethics committee and the participating school section. This single‐blinding cluster‐randomised controlled trial was conducted in April and May 2024 in a secondary school in Bursa, Türkiye, including one control and one experimental group. The study consisted of a 1‐week familiarisation period, a 4‐week intervention and an impact assessment session (Figure 1). A pretest–posttest control group design was employed.

#### 2.1.1 Control Group (CG)

CG was asked to meet their daily 10,000 steps and exercise goals using T2M mobile application. No incentives, including gamification elements (such as badges, points and leaderboards), were used to help students achieve their goals.

#### 2.1.2 Experimental Group (EG)

EG was asked to meet their daily 10,000 steps and exercise goals using the T2M mobile application. Gamification elements (such as badges, points and leaderboards) were used to help the students achieve their goals.

### 2.2 Participants

The participants were 25 secondary school students (CG: 13 and EG: 12) aged between 12 and 14 years and studying in the 7th grade (see Table 1). Inclusion criteria: 7th‐grade students, no health problems, sedentary individuals (not actively involved in sports) and getting informed consent from parents.

**TABLE 1:** Distribution of the students participating in the study according to gender.

	Control group		Experimental group	
	*f*	%	*f*	%
Gender				
Girl	9	69	8	67
Boy	4	31	4	33
Sum	13	100	12	100

*Note: f*, frequency (number of students) and %, gender distribution rate.

### 2.3 Randomisation and Blinding

Before the study began, randomisation was conducted at the class level using a computerised random number generator (cluster‐randomised). A total of four preexisting classes were included in the study. These classes were randomly assigned in a 1:1 ratio to one of two groups: the experimental group (EG) or the control group (CG). Specifically, two classes were allocated to each group. Also, single‐blinding was implemented at the participant level; students were unaware of the group allocation or the nature of the intervention assigned to the other groups.

### 2.4 T2M Mobile App Features

**FIGURE 2:**
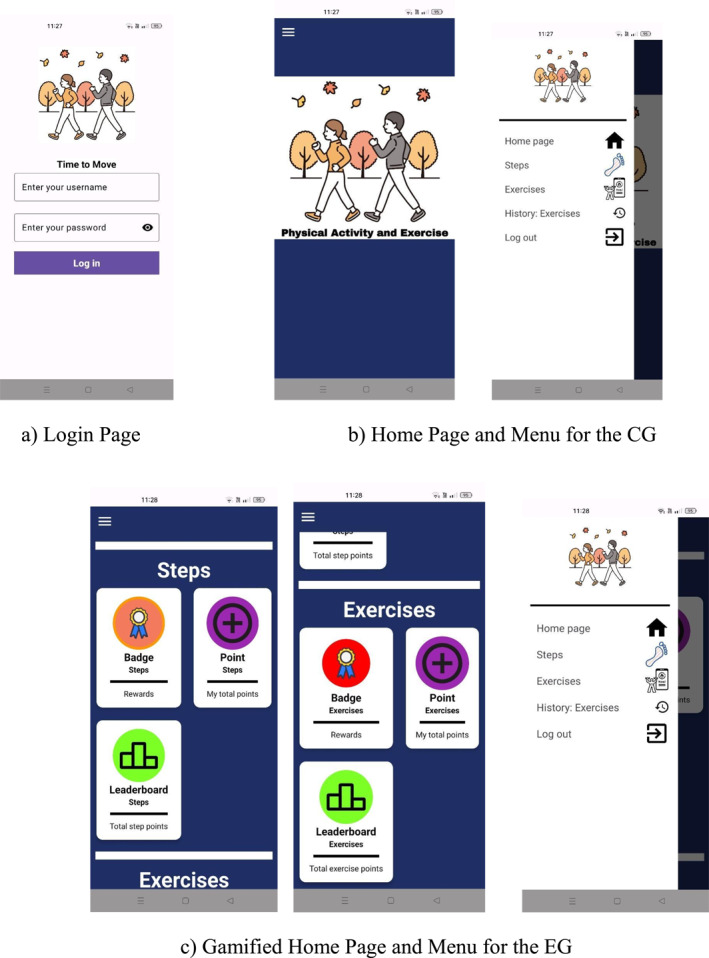
Login page (a), home page and menu for the CG (b) and gamified home page and menu for the EG (c).

T2M mobile application (Android version) was developed for students to meet their daily 10,000 steps and exercise goals and to increase their physical activity levels. Students logged into the application using their username and password (see Figure 2a). Different home pages were designed for the CG and the EG. Figure 2b (Home Page and Menu) was designed for the CG, whereas Figure 2c (Gamified Home Page and Menu) was designed for the EG. The data were stored in the ‘Firebase’ database.

#### 2.4.1 T2M Mobile App: Menu Components

The CG and EG students' daily step count and exercise behaviours were assessed using the T2M mobile‐based gamification application. The Turkish version of this mobile application was used.

**FIGURE 3:**
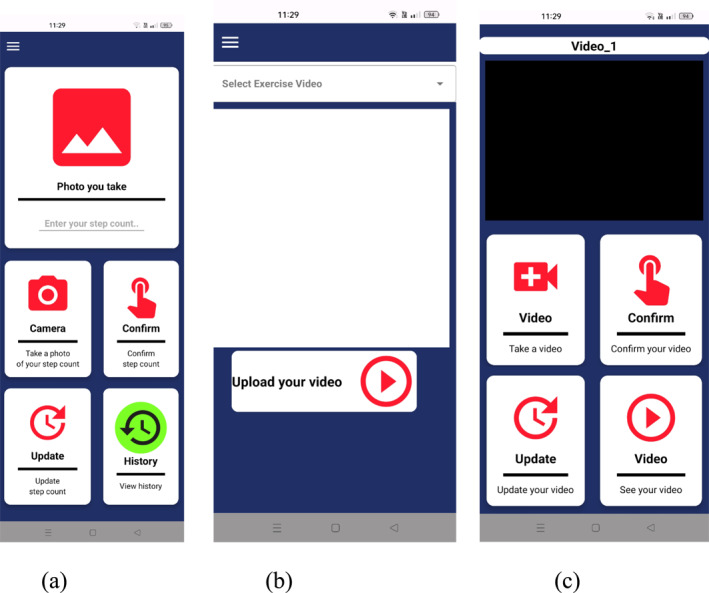
Step count recording and tracking page (a), exercise videos watching page (b) and exercise video recording and tracking page (c).

**FIGURE 4:**
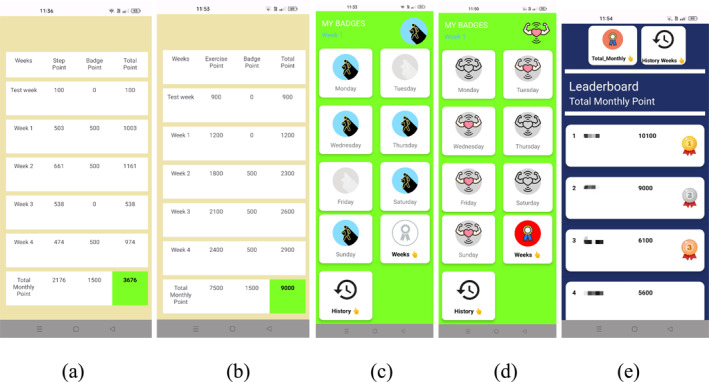
Step points (a), exercise points (b), weekly step badges (c), weekly exercise badges (d) and leaderboards (e).

Menu components (see Figure 2):

1. Steps: Recording and tracking the number of daily steps (see Figure 3a).
2. Exercises: Watching, recording and tracking daily exercise videos (see Figure 3b,c).
3. Home Page: For the EG in Figure 2c, various additional functionalities are available, including gamification elements such as (i) points, (ii) badges and (iii) leaderboard show (see Figure 4).

Figure 3a enables students to record a photo of their daily pedometer. Students can also see their past step count. In Figure 3b, students are required to complete the 10 essential exercise videos daily. They can watch the videos and record their exercise videos as shown in Figure 3.

#### 2.4.2 Design of Gamification Elements in T2M Mobile Application

##### 2.4.2.1 Step Count

100 points (one hundred steps = one point) were awarded for a daily goal of 10,000 steps. A maximum limit of 15,000 steps (150 points) was set. Even if more steps were taken, 150 points were awarded. No points were awarded if less than the daily goal of 10,000 steps were taken. A digital step badge was awarded to those who achieved the daily goal of 10,000 steps. An additional 300 points were awarded if they received at least five step badges per week.

##### 2.4.2.2 Exercise

A digital exercise badge (300 points) was awarded to those who achieved the 10 essential exercise goals daily. An additional 500 points were awarded if they received at least five exercise badges per week.

**FIGURE 5:**
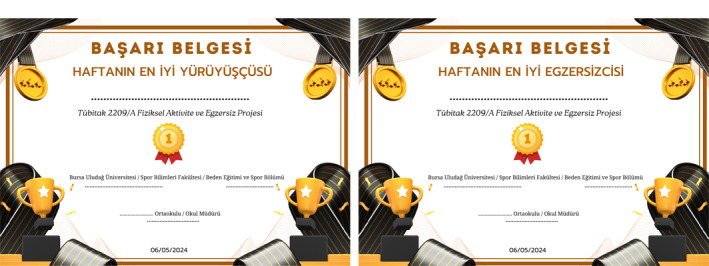
From left to right: Certificate for the best walker of the week and certificate for the best exerciser of the week.

**FIGURE 6:**
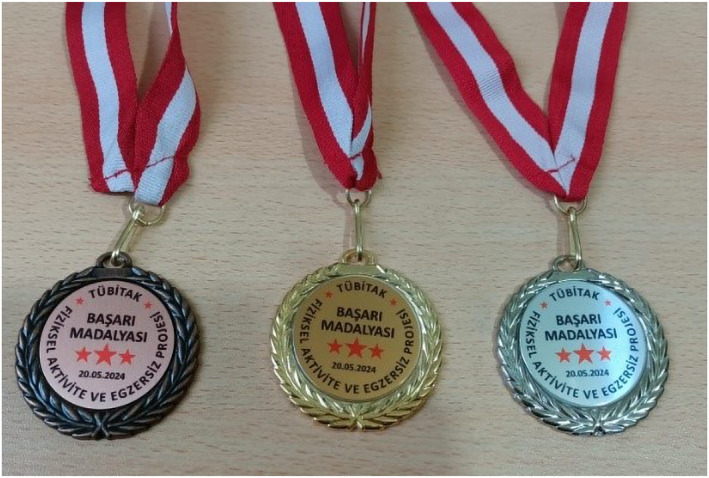
Achievement medals awarded to participants.

A student's weekly step and exercise badges are shown in Figure 4. The step count, exercise and additional points were recorded daily on the leaderboard (see Figure 4c). Only the DG received certificates and medals for achieving the step and exercise goals at the end of the study (See Figures 5 and 6). In the CG, the home page of T2M mobile application does not include the gamification design shown in Figure 2c. No intervention was applied to the CG. The CG were only asked to achieve their daily step count and exercise goals.

### 2.5 Procedure

**FIGURE 7:**
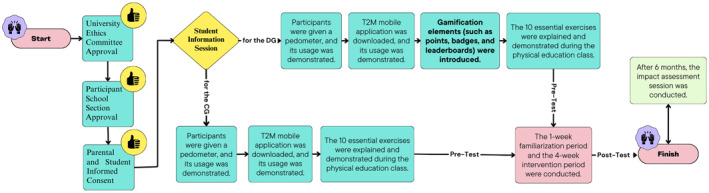
Procedure flow chart.

Separate information meetings were held to prevent cross‐contamination between CG and EG groups (See Figure 7). During these meetings, participants received pedometers and the T2M mobile application was installed on their phones. They were instructed to upload daily photos of their step counts to the mobile application each evening to ensure secure monitoring and recording. Exercises were conducted through the mobile app and recorded on video to verify adherence. This study examined the students' number of steps and exercise behaviours over 4 weeks.

### 2.6 Instruments

#### 2.6.1 Quantitative

##### 2.6.1.1 Demographic Information

Before the intervention, students answered questions on variables such as gender, age and the physical activity levels of their mother and father.

##### 2.6.1.2 Physical Activity Questionnaire for Older Children (PAQ‐C)

Students' physical activity levels were determined using the Turkish version of the PAQ‐C, which was first developed by CROCKER et al. (1997), and validity and reliability studies were conducted (Tanır 2013; Erdim et al. 2019). Currently, there are many language adaptations and modifications of the PAQ‐C (Aggio et al. 2016; Gobbi et al. 2016; Isa et al. 2019). The PAQ‐C contains questions about activity levels during physical education lessons, recess, lunch and after school activities in the evenings and on weekends. This PAQ‐C provides information about students' general physical activity habits. In calculating the physical activity scores of the participants, the average of all questions is taken. In the PAQ‐C, a score of 5 represents the highest level of physical activity, whereas a score of 1 indicates the lowest level.

##### 2.6.1.3 Step Count Tracking

Pedometers were used to track students' daily step counts and physical activity levels (Onwalk One Plus Pedometer/Accelerometer, NEWFEEL, Türkiye). Students were requested to carry the pedometer during all waking hours (except when bathing or other water‐based activities). The daily step count goal was set at 10,000 steps based on a comprehensive review by Tudor‐Locke et al. (2011). Students recorded their daily step counts using T2M mobile application. Step counts were also tracked daily through the mobile application.

##### 2.6.1.4 Exercise Tracking

The 10 essential bodyweight exercises that students were required to follow daily included four parts: warm‐up, lower body development, upper body development and cool‐down movements. The daily exercise lasted between 15 and 20 min in total. The essential exercises selected were ones that all students could perform. The exercises were demonstrated to the students in the physical education class, and videos of the exercises were also provided on the mobile application.

##### 2.6.1.5 T2M Mobile App Usage

Using the T2M mobile app, CG and EG achieved their daily goal of 10,000 step count and daily exercise. Those in the EG group earned points and badges for their tasks. At the end of the intervention, awards (certificates and medals) were given to those who succeeded in the leaderboard. Gamification elements were not included in the CG. Only step count and exercise behaviours were tracked.

#### 2.6.2 Qualitative

##### 2.6.2.1 Impact Assessment

An impact assessment session was conducted 6 months after the conclusion of the intervention to examine the effects of gamification and assess the overall impact of the study. In this session, the children were asked whether they had kept track of their step count after the intervention ended, whether they had continued doing the exercises and if so, how often. Data were collected using a paper‐and‐pencil method. This information was used to assess the impact of gamification.

### 2.7 Statistical Analysis

Quantitative data were statistically analysed using the IBM SPSS Statistics 29.0 software. The Shapiro–Wilk test showed that the data for the selected variables were abnormally distributed. In addition, the sample size was less than 30. For these reasons, nonparametric statistical methods were applied. The Mann–Whitney *U* test was used to analyse the differences between the CG and EG groups. The significance value was set at 0.05. Content analysis was used for qualitative data. Visuals were created using the matplotlib library in python. Additionally, effect sizes for group differences were expressed as Cohen's *d* (Cohen 1988); effect sizes are reported as follows: trivial (< 0.2), small (0.2–0.49), medium (0.5–0.79) and large (≥ 0.8) (Cohen 1988).

For the qualitative data, we employed an inductive content analysis approach. The first author manually conducted open coding and reviewed participant responses line by line to identify meaningful units. These codes were subsequently grouped into overarching themes reflecting students' intervention experiences. A second expert researcher independently reviewed the themes to ensure trustworthiness and disagreements were resolved through discussion.

## 3 Results

### 3.1 Quantitative Results

#### 3.1.1 Descriptive Statistics

**FIGURE 8:**
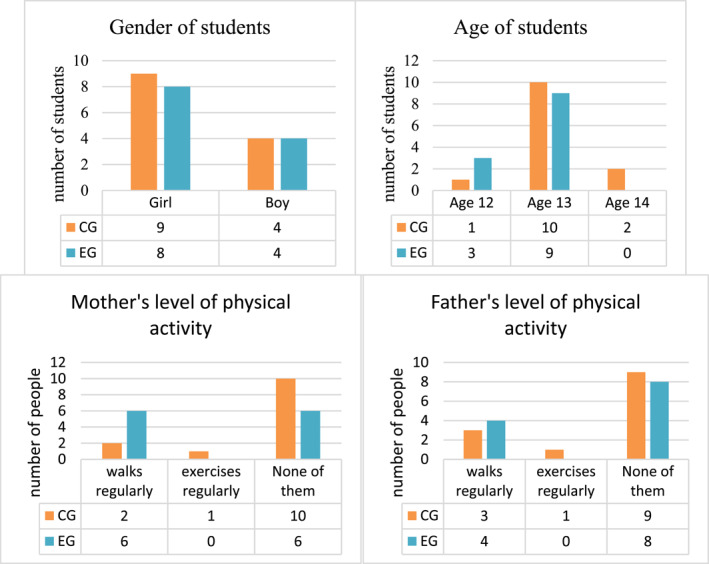
The students' demographic information forms.

The students' demographic information forms are presented in Figure 8. According to Figure 8, 17 girls and 8 boys completed the study. Additionally, 64% of the students' mothers and 68% of their fathers did not engage in physical activity.

#### 3.1.2 Research Questions

##### 3.1.2.1 Research Q1

Is there a statistically significant difference between the control group (CG) and experimental group (EG) in achieving daily 10,000 steps and exercise goals for 4 weeks?

**FIGURE 9:**
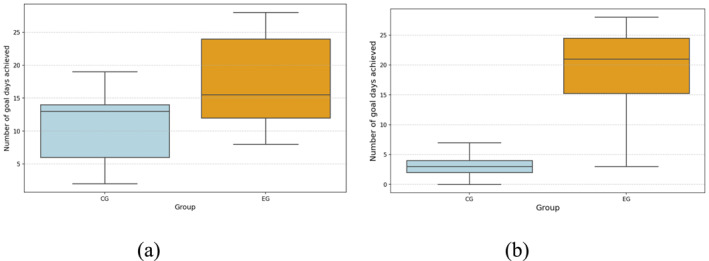
Box plot of the number of days EG and CG achieved the daily goal of 10,000 steps (a) and box plot of the number of days EG and CG achieved their daily exercise goal (b). The median value is shown for each box.

At the end of the 4‐week intervention period, the CG exercised on an average of 4 days, whereas the EG exercised on an average of 19 days. The CG achieved the 10,000 steps goal on average in 11 days, whereas the EG achieved the 10,000 steps goal on average in 18 days (See Table 2). The number of days the EG achieved 10,000 daily steps and exercise goals was higher than CG (See Figure 9). As a result, it was observed that the EG completed more daily steps and exercise goals. In addition, the Mann–Whitney *U* test results revealed a statistically significant difference between the CG and EG groups in achieving the daily 10,000 steps and daily exercise goals for 4 weeks (step goal: *U* = 115.5, ***p* = 0.043** and ***d* = 0.40** and exercise goal: *U* = 149.0, ***p* = 0.0001** and ***d* = 0.77**).

**TABLE 2:** Comparison of physical activity outcomes between control and experimental groups.

Outcome measure	Control group (CG)	Experimental group (EG)
Average days achieving exercise	4 days	19 days
Average days achieving 10,000 steps	11 days	18 days
PAQ‐C (pretest)	2.2	2.4
PAQ‐C (posttest)	2.5	3.4

**FIGURE 10:**
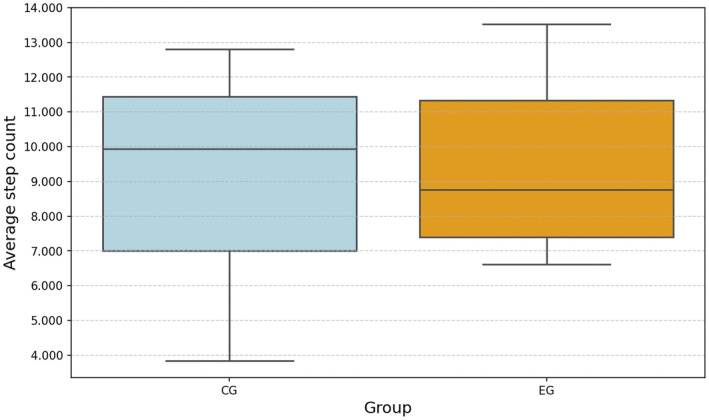
Average step counts for EG and CG. The median value is shown for each box.

At the end of the study, EG participants took an average of 9493 steps, whereas CG participants took an average of 9185 steps (See Figure 10).

##### 3.1.2.2 Research Q2

Is there a statistically significant difference between pretest and posttest results of the Physical Activity Questionnaire for Older Children (PAQ‐C) in the control group (CG) and experimental group (EG)?

**FIGURE 11:**
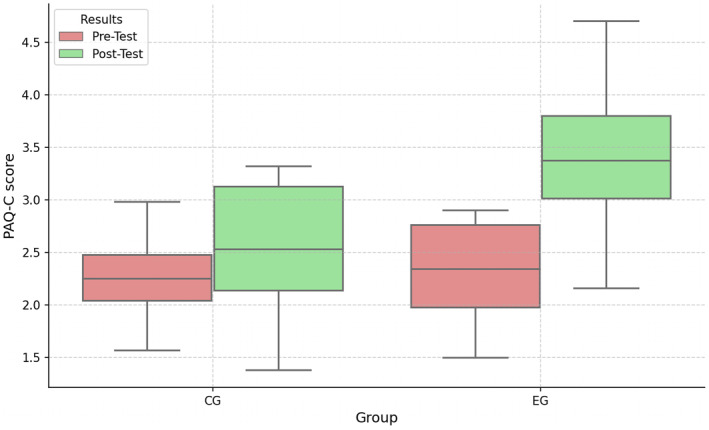
PAQ‐C pretest and posttest results for the CG and EG.

Although the average physical activity level of CG increased from 2.2 to 2.5, that of EG increased from 2.4 to 3.4. Additionally, the physical activity level of EG showed a greater increase (See Figure 11). In addition, according to the statistical analysis results, a statistically significant difference was found between the pretest and posttest of EG and CG groups (*U* = 125.0, ***p* = 0.0114 and *d* = 0.51**).

### 3.2 Qualitative Results

#### 3.2.1 Impact Assessment

The responses were analysed to determine whether the students used pedometers and continued their exercise behaviours.

**FIGURE 12:**
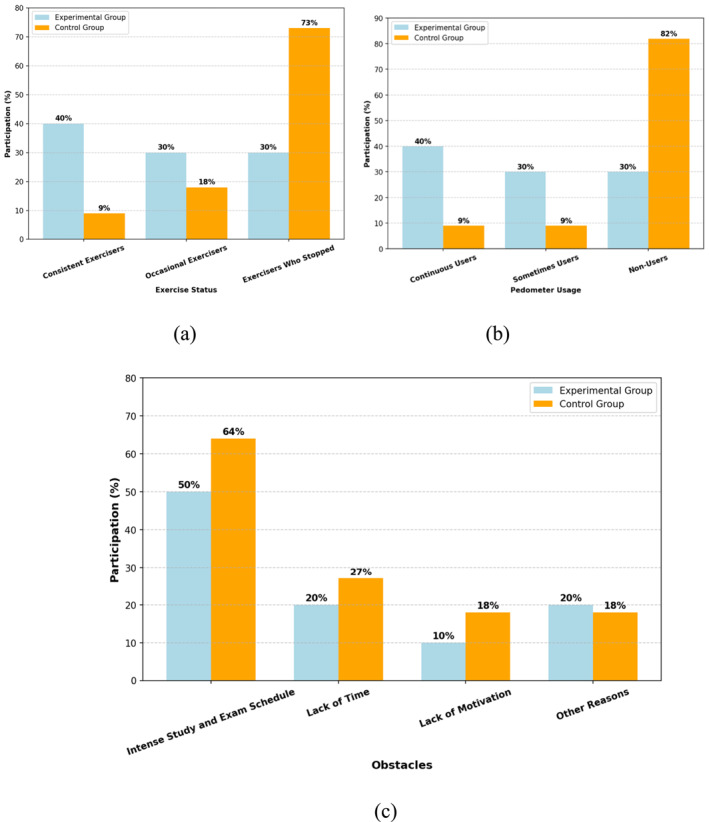
Comparison of continued exercisers and quitters (a), comparison of pedometer usage and tracking in the EG and the CG (b) and factors that hinder exercise (c).

Although 70% of the EG continued to exercise, only 27% of the CG continued to exercise (See Figure 12a). EG exercised significantly more than CG. Although 70% of the EG used the pedometer continuously or sometimes, this rate was only 18% in the CG (See Figure 12b). The intensity of the study and exam schedule was the primary obstacle reported by both groups (See Figure 12c).

## 4 Discussion

The aim of this study was to examine the association between gamification of physical activity and exercise and physical activity levels in secondary school students. During the intervention, 40% of those in the control group completed the step goals and 15% completed the exercise goals. In comparison, 65% of the experimental group completed the step goals and 68% completed the exercise goals. Furthermore, the PAQ‐C scores were found to be higher in the experimental group. As a result of the impact assessment session, it was observed that 70% of the students in the experimental group and 27% of those in the control group continued to exercise. It is seen that the students in the experimental group were more likely to use pedometers than the control group and continued their exercise behaviour more than the control group. In addition, it is seen that the intensity of lessons and exams is the most significant factor associated with reduced exercise for both groups. Our findings suggest that T2M, a mobile application gamified by physical activity and exercise, is acceptable to children and adolescents. It is seen that the experimental group was effective in achieving their daily step count and daily exercise goals with the use of the T2M gamification mobile application.

Our findings support the results of previous studies. For example, Willinger et al. (2024) examined the ‘KIJANI’ gamified step count mobile application, which was specifically developed for children and adolescents and rewards step counts with points. Their findings suggest that the application was associated with higher physical activity levels. Similarly, other gamified mobile applications for step counting, such as Pokémon Go, Pacer, Strava and MapMyWalk, were used in a 10‐week intervention. Results showed that these applications were linked to increased physical activity levels (Mateo‐Orcajada et al. 2023). In addition, 107 students aged 12–13 years reported significant increases in their daily steps in a study involving only the experimental group (Vorlíček et al. 2024).

In contrast to our findings, a 72 h field study by Ahn et al. (2019) found that a point‐based reward system only temporarily increased physical activity (PA) engagement but did not significantly affect overall PA levels over time in children aged 9–13 years (point group: *n* = 39 and nonpoint group: *n* = 27). This study utilised a single gamification element—the point‐based reward system—to promote PA among children. However, our findings suggest that incorporating additional gamification elements, such as badges and leaderboards, alongside a point‐based reward system is associated with higher physical activity levels.

In general, studies have focused on the gamification of pedometer use. However, our study also included an exercise intervention. As our results show, gamification was associated with increased exercise behaviour. To support this, Wong et al. (2020) designed a mobile application called ‘Family Move’ to enable children and parents to exercise together. In this application, users earned points as they completed exercise videos, and it was reported that those who collected at least 5000 points during the intervention period (8 weeks) would receive a cash prize. As a result, gamification may be associated with increased exercise behaviour.

The literature shows that studies on pedometer intervention only or exercise only in children and adolescents are limited. Therefore, when similar studies conducted in different age groups are examined, it is seen that there are mobile or internet‐based interventions to promote PA in adults or the elderly. For adults, these are the ‘WeChat’ based intervention of Mo et al. (2019), which integrates gamification and social incentives, and the gamified pedometer mobile applications in Table 3. In addition, a comprehensive exercise gamification application for adults (running, walking and strength exercises) is seen in Sporrel et al. (2021) ‘PAUL’ application and Olivas Martinez et al. (2023) ‘Push Up Game’ (PUG) mobile gamification application. In all of these studies, it is seen that physical activity levels were higher with the use of gamification elements. According to a meta‐analysis conducted by Mazeas et al. (2021), gamification enhances participation in physical activity more effectively than traditional nongamified interventions.

**TABLE 3:** Gamified pedometer mobile applications.

Participants	Mobil app name	Authors
Adult	Just walk	Korinek et al. (2017)
Adult	StepByStep	Zuckerman and Gal‐Oz (2014)
Elder	ActivityCoach	Tabak et al. (2020)
Elder	BAND	J. Lee and Ryu (2023)

The existing literature's pedometer and exercise gamification interventions were not explicitly designed for children and adolescents. In contrast, the study by Edney et al. (2017) developed the ‘ACTIVE TEAM’ mobile application, which shares similarities with our study but targets individuals aged 18–65. This app encouraged participants to take 10,000 steps daily and engage in at least 150 min of moderate or vigorous physical activity per week. A key difference from our application is that it includes notifications and social support features via Facebook. This study demonstrates that gamification is associated with increased physical activity levels, supporting the findings of our study. Similarly, Goh and Razikin (2015) demonstrated that social support features, such as liking, commenting and following, are important for motivating individuals to exercise. Our study suggests that incorporating social support features could inspire new research.

### 4.1 Strengths and Limitations

The present study had several strengths that should be mentioned: (i) Automatic scoring and tracking of daily step counts and exercises from our T2M mobile app; (ii) single‐blinding cluster‐randomised controlled trial and (iii) data accuracy can be verified through the T2M mobile app. On the other hand, certain limitations should also be acknowledged: (i) Lack of G‐Power analysis; (ii) unequal gender distribution; (iii) 4‐week intervention period and (iv) relatively small number of participants (25 students), may limit the generalisability of the findings.

## 5 Conclusion

In conclusion, to the best of our knowledge, our study is the first to evaluate the effects of a gamification‐based mobile application intervention that combines game design elements (points, badges and leaderboards) with step count and exercise on physical activity levels in children and adolescents. Our findings suggest that sedentary children and adolescents can increase their physical activity levels by using a gamified mobile application. However, the sample size of this study is limited; therefore, future research should involve a larger group of participants to assess the effects of mobile‐based gamification interventions better. Furthermore, it is recommended that children and adolescents use T2M to promote physical activity in out‐of‐school environments.

## Conflicts of Interest

The authors declare no conflicts of interest.
